# Memory Problems During COVID in Low-income Older Adults

**DOI:** 10.1093/geroni/igab046.2739

**Published:** 2021-12-17

**Authors:** Faika Zanjani, Annie Rhodes

**Affiliations:** Virginia Commonwealth University, Richmond, Virginia, United States

## Abstract

Prevention, with widespread lifestyle risk reduction at the community-level, is currently considered an effective method to decrease Alzheimer’s disease (AD). As part of the Virginia Commonwealth University iCubed Health and Wellness in Aging Core, diverse older adults (60+) living in Richmond, VA, with incomes below $12,000/year and managing either diabetes/cardiovascular symptoms, were offered weekly lifestyle telephone-health coaching for 12-weeks, providing education, motivations, self-efficacy, and referral services for AD lifestyle risk. The study sample (n=40, mean age 68 years (range: 60-77 years) was 88% African American/Black (n=35), 100% Non-Hispanic, and 45% males (n=18)). Thirty-nine (95%) of subjects successfully participated in coaching sessions; on average 91.9% (11) sessions/subject were completed. Participants provided positive anecdotal feedback and the need for continued health coaching during COVID. N=30 (75%) of the original sample consented for continued health coaching during the Covid pandemic, 63% female, 88% African American/Black, 60-77 age range (mean age 69 years), and 47% reporting memory problems. Baseline Covid interviews indicated poorer health status associated with reporting memory problems for poor physical health days (F=7.03;p=.01); poor mental health days (F=6.88;p=.01); total mental/physical health poor days (F=2.76;p=.11); sad days (F=15.52;p=.001); worried days (F=6.27;p=.02); tired days (F=9.77;p=.004); feelings of emptiness (F=10.09;p=.004); feelings of rejection (F=3.382;p=.08); feelings of failure (F=7.58;p=.01); little interest/pleasure (F=7.84;p=.009); and feeling down (F=6.75;p=.02). In conclusion, this preliminary work creates the impetus for future large-scale AD prevention investigations to improve the lives of AD-risk, low-income, diverse older adults reporting memory problems. This research indicates the subjective reporting of memory problems requires health intervention.

